# Pica in a girl with non-suicidal self-injury: a case report

**DOI:** 10.3389/fpsyt.2023.1320079

**Published:** 2023-12-18

**Authors:** Bo Liu, Lijun Jiang, Minlan Yuan, Hongru Zhu, Wei Zhang

**Affiliations:** ^1^Mental Health Center and Psychiatric Laboratory, West China Hospital of Sichuan University, Chengdu, China; ^2^Zigong Mental Health Center, Zigong, China; ^3^West China Biomedical Big Data Center, West China Hospital of Sichuan University, Chengdu, China

**Keywords:** pica, NSSI, MECT, CBT, quetiapine, lithium, sertraline

## Abstract

Non-suicidal self-injury (NSSI) is on the rise globally, posing a significant societal challenge. Pica, an eating disorder, presents difficulties in treatment due to the absence of effective medications. In this report, we discuss a complex case involving the co-occurrence of pica and non-suicidal self-injury. A 13-year-old girl was admitted to our hospital due to ingesting two batteries. She features a persistent, intense appetite along with sudden and compulsive behaviors such as consuming inedible items or self-inflicted cutting. After receiving a combination of pharmacological treatments (quetiapine, lithium and sertraline), cognitive behavioral therapy (CBT) and modified electroconvulsive therapy (MECT) for 25 days, she was discharged with relief from her clinical symptoms.

## Background

Pica is characterized by the compulsive consumption or intense craving of non-food, non-nutritive substances (1). It can either be a distinct mental disorder or manifest as a clinical symptom of other diseases, such as autism spectrum disorders, obsessive compulsive disorder, or schizophrenia. According to the Diagnostic and Statistical Manual of Mental Disorders, 5th Edition (DSM-5), pica must endure for over 1 month at an age where consuming non-food items is developmentally inappropriate, not culturally accepted, and of a severity that necessitates clinical intervention (2). Pica is most commonly observed in pregnant women (3) and young children, and while some cases are short-lived, others can persist for an extended duration. Lesinskienė et al. found that around 3.7% of 614 participants reported instances where their child consumed non-nutritious, non-food substances (4). There are reports suggesting that pica may be linked to deficiencies in minerals (e.g., iron, zinc) (5–7). However, the precise pathophysiology of pica remains unclear. Non-suicidal self-injury (NSSI) is characterized by purposeful harm inflicted upon one’s own body tissues, with no intent of suicide and involves actions that are not socially accepted (8). Common behaviors associated with NSSI include actions like cutting, burning, biting, and scratching the skin. A meta-analysis involving 686,672 children and adolescents, the overall lifetime prevalence of NSSI was 22.1%, while the prevalence within the past 12 months was 19.5% (9). Another meta-analysis reported that NSSI behavior impacts millions of adolescents annually, underscoring a pressing public health issue that requires attention, research, and treatment (10).

## Case presentation

Miss A, a 13-year-old girl, was admitted due to 2 years of persistent self-injury and ingesting non-food substances. Two years ago, she began to exhibit recurrent arm cutting. She was excited to see the blood but had no intention of committing suicide. Frequent arm cuts left extensive scars on her arms. Subsequently, she began swallowing inedible substances such as a nail clipper, pen, battery, clay, and shampoo. When she experiences a strong urge to swallow, she cannot resist looking for anything to ingest. Miss A knows that self-injury and ingesting inedible objects are inappropriate and harmful, but she cannot control herself. It’s interesting to note that she appeared unconcerned about her health and did not exhibit significant symptoms of anxiety or depression during the inter-episode period. The severity of her pica and self-injury behaviors has led to her frequent hospitalization. The emotional and behavioral abnormalities in the case occur episodically, impulsively, and irregularly, without the presence of constant mood disorders. We present a mini-interview with the patient (see Table 1).

**Table 1:** A mini-interview with the patient.

	Questions and answer
Doctor	Why do you frequently hurt yourself?
Patient	I do not know, just feel an uncontrollable and sudden urge to do it.
Doctor	When or in what situations do you usually hurt yourself?
Patient	I tend to do it when feeling down or bored.
Doctor	How do you usually feel after swallowing objects or self-injury?
Patient	I feel pleased and even excited, especially when seeing blood.
Doctor	Are you aware of the consequences of your actions?
Patient	Maybe die
Doctor	Do you have thoughts of suicide?
Patient	No
Doctor	How do you perceive or evaluate your risky behaviors?
Patient	Nothing matters

Miss A has experienced normal physical and mental development, as she has no prior medical history and had a good academic performance before the start of her symptoms. She has been living with her grandparents since her parents divorced when she was one-year old. Her father doted on her and never remarried. Miss A initially interacted externally and enjoyed playing with students, but later became more withdrawn, spending less time with students and engaging in online gaming until midnight. She also experienced impaired attention and had poor academic performance due to her illness, which led her to drop out of school for 1 year. In addition, she experienced a significant increase in appetite, often seeking food and becoming less physically active. This led to a weight gain from 45 kg to 65 kg, resulting in BMIs of 17.15 and 24.76, respectively.

**Figure 1:**
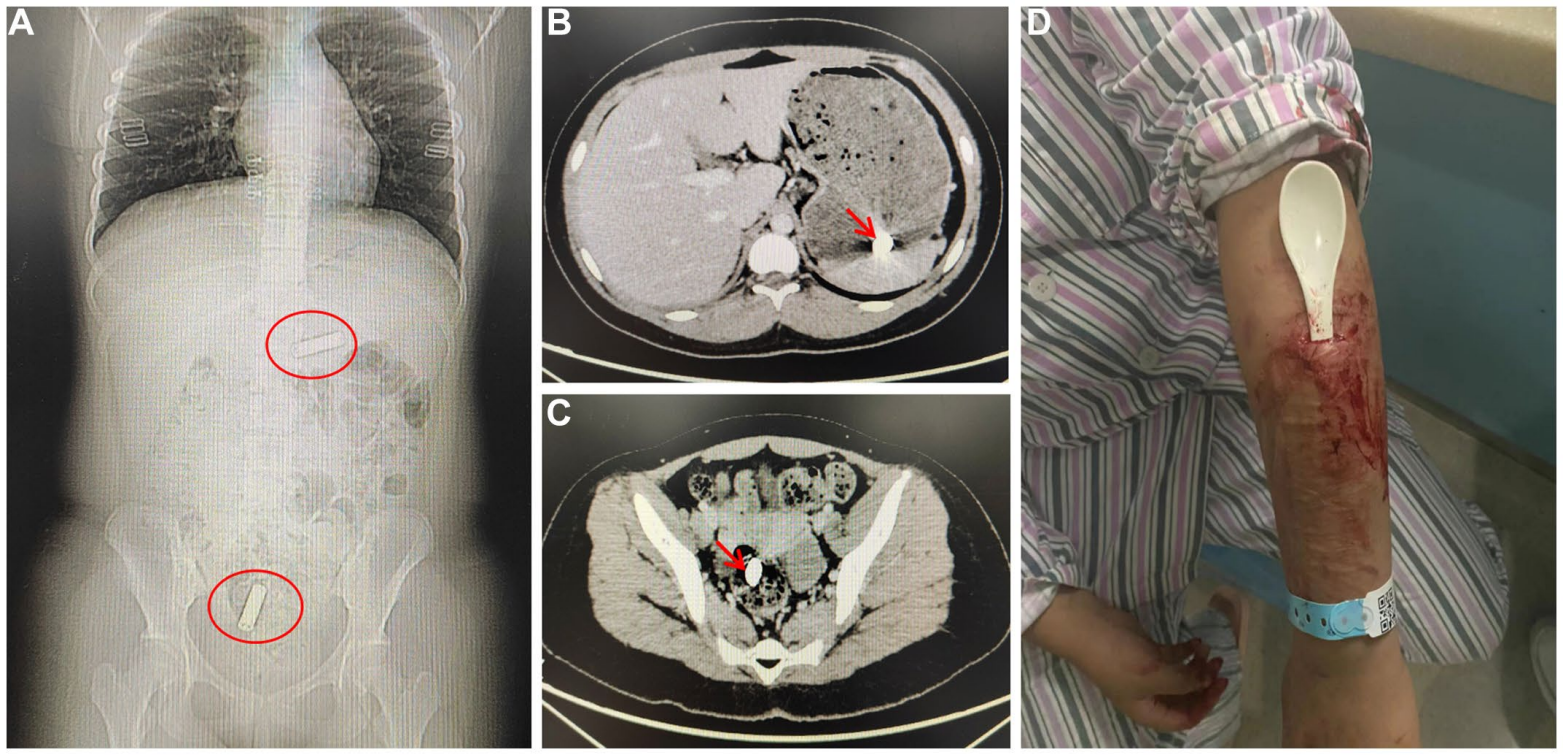
**(A)** CT scan shows the position of the batteries in red circle; **(B)** One battery in the stomach; **(C)** One battery in the colon; **(D)** An image shows a plastic spoon inserted into her left arm.

The laboratory tests did not reveal any other abnormal findings, such as anemia, lack of vitamin or minor element deficiencies. However, a computerized tomography scan detected the presence of two batteries in her stomach and intestines (Figures 1A–C). The batteries passed through her digestive track and were expelled during a bowel movement within 2 days. While in the hospital, she consumed a bottle of toner, necessitating a gastric lavage procedure. Subsequently, she inserted a plastic spoon into her arm, requiring debridement and suturing treatment (Figure 1D). Furthermore, she underwent modified electroconvulsive therapy (MECT) six times and was prescribed polypharmacy, including quetiapine, lithium and sertraline, in conjunction with cognitive behavioral therapy (CBT). Throughout the last week in the hospital, there were no instances of self-injury or swallowing of objects observed. She was discharged with significant clinical relief and proceeded with outpatient follow-up. After 2 weeks, she continued with the prescribed pharmacological treatment and remained in good condition. You can find the comprehensive treatment process outlined in Table 2.

**Table 2:** A timetable of combined treatments.

	Day0	Day1	Day5	Day10	Day15	Day20	Day25	2w later
Sertraline	150 mg qd	150 mg qd	100 mg qd	100 mg qd	100 mg qd	50 mg qd	50 mg qd	50 mg qd
Lithium	250 mg bid	250 mg bid	250 mg tid	500 mg bid	250 mg tid	250 mg bid	250 mg bid	250 mg bid
Olanzapine	2.5 mg qn	2.5 mg qn	5 mg qn	×	×	×	×	×
Lorazepam	×	0.5 mg tid	0.5 mg tid	0.5 mg bid	0.5 mg qn	×	×	×
Quetiapine	×	×	×	50 mg qn	100 mg qn	200 mg qn	200 mg qn	200 mg qn
CBT	×	√	√	√	√	√	√	×
MECT	×	×	×	×	√	√	×	×

CBT, cognitive behavioral therapy; MECT, modified electroconvulsive therapy; ×, no application; √, treatment; Day0, before admission; Day1, the day of admission; Day25, the day of discharge; 2w later, 2 weeks after discharge.

## Discussion

While the co-occurrence of NSSI and eating disorders is common (11), this is the first report, to our knowledge, of pica and non-suicidal self-injury in children. The case presents with two groups of syndromes: eating disorders and emotional and behavioral syndromes. This case is characterized by a persistent increase in appetite, alongside intermittent emotional and behavioral symptoms such as dysphoria, anger outbursts, consuming non-nutritive items, and self-harm by cutting her arms.

The precise cause of Pica remains largely unknown. Pica has been shown to be associated with various causes, including lack of vitamin (12) or minor element deficiencies (13), neurodevelopmental diseases (14), and other mental disorders (15). Pica-symptoms can range from mild and self-limited to potentially life-threatening and long-lasting. In clinical practice, the treatment of pica primarily focuses on managing complications, including infection, poisoning, intestinal obstruction, and severe anemia. The World Federation of Societies of Biological Psychiatry (WFSBP) guidelines update 2023 on the pharmacological treatment of eating disorders has summarized reports of the pharmacological treatment of pica (16). So far, case reports in pica document the use of antipsychotics (the atypical antipsychotics olanzapine, paliperidone, risperidone, and aripiprazole), antidepressants (the SSRIs escitalopram, fluoxetine, fluvoxamine, and paroxetine), mood stabilizers (lithium and topiramate), benzodiazepines (clonazepam) and stimulants (methylphenidate). However, no randomized-controlled pharmacological trials (RCT) have been published so far. As care reports are considered low evidence, the WFSBP guidelines update did not make strong recommendations for or against the use of these medications (16). In contrast, behavioral treatments show more promise as a potent means of preventing pica (17). Typical interventions for pica often involve response blocking, differential reinforcement of alternative behavior (DRA), response interruption and redirection (RIRD), and noncontingent reinforcement using competing stimuli (18). In line with earlier findings, her impulsive self-injury behaviors are directed towards either alleviating dysphoria or seeking pleasure (19). These inappropriate behaviors are typically associated with unstable emotions resulting from hedonism (20). NSSI has been shown to correlate with an increased risk of future suicidal behaviors (9). The presence and quantity of NSSI scars serve as objective physical indicators of the risk for suicidal behaviors (21).

The efficacy of antidepressants in addressing NSSI remains a subject of controversy, while atypical antipsychotics have shown efficacy in improving impulse control and behavior (11). Hence, we reduced the dosage of sertraline while simultaneously increasing the dosage of olanzapine. Due to the heightened appetite associated with olanzapine, we substituted olanzapine with quetiapine. Lithium is a mood-stabilizing drug commonly employed in the treatment of bipolar disorder. There are reports suggesting that lithium can reduce suicidal ideation and NSSI in adolescents within a residential treatment center (11). MECT is a highly effective treatment for severe depression or mania; however, there are few reports of its use in treating NSSI in children. Considering her strong urge to ingest non-edible items and engage in self-injury, we conducted a comprehensive evaluation of her condition and determined that the use of MECT was appropriate. In addition, while there are currently no psychotherapy treatments specifically designed and evaluated for children and adolescents engaging in clinically significant levels of NSSI, cognitive and behavioral interventions hold the most promise for providing therapy to them with NSSI (22). However, it’s worth noting that the combination of multiple medications may increase the risk of adverse drug reactions (ADRs). We monitored her liver and renal function and electrocardiography one a week during hospitalization and no significant ADRs were detected.

From a psychosocial perspective, the population of children engaging in NSSI is rapidly increasing in China, observed in both mental health institutions and schools. Children often experience a high standard of living provided by their parents, along with the natural human desire for happiness. This situation can create a lack of necessary live challenges in their growth, causing their happiness to be reliant on external sources rather than stemming from within. When they are unable to obtain what they desire from others, they may easily become prone to anger or depression. Hence, altering our way of life, curbing our desires, and enabling children to evolve at their own pace could be a viable strategy to tackle the issue of children engaging in NSSI.

## Conclusion

In summary, we show a rare case of pica complicated with NSSI in a girl. Furthermore, we provide a promising combination of multiple medications, psychotherapy, and physiotherapy. However, we have to admit that this is a complicated case, involving multiple potential bio-psycho-social factors. Much more attention is needed for long-lasting follow-up.

## Data availability statement

The original contributions presented in the study are included in the article/supplementary material, further inquiries can be directed to the corresponding author.

## Ethics statement

Written informed consent was obtained from the individual(s), and minor(s)’ legal guardian/next of kin, for the publication of any potentially identifiable images or data included in this article.

## Author contributions

BL: Writing – original draft. LJ: Writing – review & editing. MY: Writing – review & editing. HZ: Writing – review & editing. WZ: Conceptualization, Supervision, Writing – review & editing.
